# Evidence for Gating Roles of Protein Kinase A and Protein Kinase C in Estradiol-Induced Luteinizing Hormone Receptor (*lhcgr*) Expression in Zebrafish Ovarian Follicle Cells

**DOI:** 10.1371/journal.pone.0062524

**Published:** 2013-05-03

**Authors:** Ka-Cheuk Liu, Wei Ge

**Affiliations:** School of Life Sciences and Centre for Cell and Developmental Biology, The Chinese University of Hong Kong, Shatin, New Territories, Hong Kong, China; Clermont Université, France

## Abstract

Estradiol (E2) stimulates luteinizing hormone receptor (*lhcgr*) expression in zebrafish follicle cells via nuclear estrogen receptors (nERs) that are likely expressed on the membrane, and *lhcgr* responds to E2 in a biphasic manner during 24-h treatment. These observations raise an interesting question on the signaling mechanism underlying E2 regulation, in particular the biphasic response of *lhcgr* expression. In the present study, we demonstrated that E2 regulation of *lhcgr* was significantly influenced by the activity of cAMP-PKA pathway. Activation of cAMP-PKA pathway by forskolin or db-cAMP suppressed E2-stimulated *lhcgr* expression in short-term (3 h) but enhanced its effect in long-term (24 h), suggesting differential roles of PKA at these two phases of *lhcgr* response. PKA inhibitor H89 showed reversed effects. In contrast, PKC pathway had consistent permissive effect on E2-induced *lhcgr* expression as evidenced by strong inhibition of E2 effect by PKC inhibitors GF109203X and Ro-31-8220 at both 3 and 24 h. One of the mechanisms by which PKA and PKC gated E2 effect might be through regulating nERs, particularly *esr2a*. Despite the strong influence of PKA and PKC, our data did not suggest direct mediating roles for these two pathways in E2 stimulation of *lhcgr* expression; yet they likely play critical gating roles in E2 signal transduction. As a follow-up study to our previous report on E2 regulation of gonadotropin receptors in the zebrafish ovary, the present study provides further evidence for the involvement of classical intracellular signal transduction pathways in E2 stimulation of *lhcgr* expression in the follicle cells.

## Introduction

Follicle-stimulating hormone (FSH) and luteinizing hormone (LH) are gonadotropins (GTHs) that signal through their cognate receptors, FSH receptor (FSHR) and LH/choriogonadotropin receptor (LHCGR), to control major gonadal events in vertebrates, including folliculogenesis and steroidogenesis in the ovary [1], [2]. The expression levels of FSHR and LHCGR in the somatic follicle cells (granulosa and theca cells), therefore, determine the responsiveness of ovarian follicles to GTHs and hence govern the development and function of the ovary.

We have recently demonstrated distinct expression profiles of zebrafish *fshr* and *lhcgr* during folliculogenesis, which showed an earlier increase in *fshr* expression and a delayed expression of *lhcgr* [3], [4]. This temporal difference in expression between *fshr* and *lhcgr* has raised a question on the control of these receptors in the zebrafish ovary. Although studies on expression control of gonadotropin receptors (GTHRs) in teleosts are increasing, the information still remains scarce compared with that in mammals. FSH has been reported to regulate GTHRs differentially by reducing *fshr* but promoting *lhcgr* expression in the coho salmon [5]. In the Japanese eel, *in vivo* treatment with pituitary extract stimulated both *fshr* and *lhcgr* expression in the ovary [6] whereas both receptors showed increased expression in the black porgy after injection with E2 [7]. We recently reported that bone morphogenetic protein (BMP) family and epidermal growth factor (EGF) family might also be involved in the regulation of GTHRs in the zebrafish. BMP members Bmp2b and Bmp4 differentially reduced *fshr* but stimulated *lhcgr* expression [8]. In contrast, EGF strongly suppressed E2-stimulated *lhcgr* expression while enhancing *fshr* expression. Other members of EGF family, including heparin-binding EGF-like factor (Hbegf), transforming growth factor α (Tgfa) and betacellulin (Btc), also showed similar inhibitory effects on *lhcgr* expression [9].

In addition to the growth factors, we have also reported differential regulation of *fshr* and *lhcgr* by gonadal steroids in the zebrafish ovary. E2 stimulated both *fshr* and *lhcgr* expression in cultured zebrafish follicle cells; however, the potency of E2 action on *lhcgr* expression was much higher than that on *fshr* expression. Interestingly, the response of *lhcgr* expression to E2 exhibited a unique biphasic pattern during a 24-h treatment period. The expression increased quickly in response to E2 treatment and the level reached the peak at 1.5 to 3 h of treatment. This was followed by a steady decline of *lhcgr* expression with the trough reached at around 6 h. However, the expression rebounded at 12 h, reaching a second peak of response at 24 h. Both phases of response were dependent on transcription but not translation and involved nuclear estrogen receptors (nERs) that appeared to be located on the plasma membrane of the follicle cells [10]. This raises an interesting question about the intracellular signaling mechanisms underlying the action of E2, especially its biphasic effects on *lhcgr* expression. Our early study provided evidence for modulatory roles of both p38 MAPK and MAPK3/1 pathways in enhancing E2 stimulation of *lhcgr* expression [10]. This points to the possibility that the E2 stimulation of *lhcgr* expression and the action of nERs might be mediated or modulated by other intracellular signaling pathways as well. To test this hypothesis, we carried out the current study to examine how activation or inhibition of cAMP-PKA and PKC pathways would influence the biphasic effects of E2 on *lhcgr* expression in cultured ovarian follicle cells at 3 h (short-term) and 24 h (long-term).

## Materials and Methods

### Animals

Adult zebrafish (*Danio rerio*) were maintained in flow-through aquaria of 60 L at 28 C under 14L∶10D photoperiod control. All fish were fed three times a day with the tropical fish feed Otohime S1 (Marubeni Nisshin Feed Co., Tokyo, Japan). All experiments were performed under a license from the Government of the Hong Kong Special Administrative Region [Ref No.: (11–116) in DH/HA&P/8/2/1 Pt.17] and endorsed by the Animal Experimentation Ethics Committee of the Chinese University of Hong Kong.

### Hormones and chemicals

All common chemicals were obtained from Sigma-Aldrich (St. Louis, MO), USB Corporation (Cleveland, OH), GE Healthcare (Waukesha, WI), or Merck (Whitehouse Station, NJ). 17β-estradiol (E2; Sigma-Aldrich) was first dissolved in absolute ethanol as stock. H89, GF109203X, Ro-31-8220 and phorbol 12-myristate 13-acetate (PMA) were purchased from Calbiochem (La Jolla, CA) and prepared in dimethyl sulphoxide (DMSO). Forskolin and dibutyryl cAMP (db-cAMP) were purchased from Sigma-Aldrich and dissolved in DMSO and water respectively.

### Primary cell culture and drug treatment

The primary cell culture and the experimental scheme of drug treatments were based on our previous report [10]. Briefly, ovarian follicles were cultured in M199 (Gibco-BRL, Gaithersburg, MD) with 10% FBS (Hyclone, Logan, UT) for six days to proliferate the somatic follicle cells. The follicle cells were then trypsinized and subcultured into 24-well plates at density of 2×10^5^ cells per well. After 24-h subculture for cell attachment, the medium was changed and the cells were starved in M199 without FBS for another 24 h. Treatments were carried out during the following 24-h time frame after the starvation as described in our recent report [10]. All cells were therefore incubated for the same period of time.

### Total RNA extraction and real-time qPCR

The number of cells in each culture well was strictly controlled and the entire RNA from each well was extracted with TRI Reagent (Molecular Research Center, Cincinnati, OH) according to manufacturer's protocol and used for RT reaction to obtain cDNA by M-MLV reverse transcriptase (Invitrogen, Grand Island, NY). Real-time qPCR was performed on C1000 Thermal Cycler CFX96 Real-time PCR Detection System (Bio-Rad, Hercules, CA) using primers listed in our previous report [10].

### Fractionation of follicle cells

The cytosol and membrane fractions of follicle cells were separated by Qproteome Cell Compartment Kit (Qiagen, Düsseldorf, Germany) according to the company's protocol. Briefly, the follicle cells cultured in a 60-mm cell culture dish were washed with ice-cold PBS twice. The cells were collected with a cell scraper and transferred to a microtube. The cells were then lysed and incubated with Buffer CE1 at 4 C followed by centrifugation at 1000×g to obtain the cytosol fraction from the supernatant. Further incubation of cell lysate with Buffer CE2 at 4 C and centrifugation at 6000×g resulted in concentrated membrane proteins in the supernatant. The extracted proteins from cytosol and membrane fractions were then precipitated by acetone and resuspended in SDS sample buffer for Western blot analysis.

### Western blot analysis

Western blot analysis was carried out according to our previous report [10]. Briefly, the cultured follicle cells were lysed by SDS sample buffer [100 µl per well; 62.5 mM Tris-HCl (pH 6.8), 1% w/v SDS, 10% glycerol, and 5% 2-mercaptoethanol]. The lysate was transferred to a microtube and heated at 95°C for 10 min. The heated samples (10 µl) and the biotinylated protein ladder (Cell Signaling Technology, Danvers, MA) were stacked by a 4% SDS gel and resolved by a 12% SDS gel by electrophoresis. The resolved proteins were transferred to a PVDF membrane (Bio-Rad). After blocking with 5% milk for 1 h, the membrane was incubated with phospho-CREB (#9191), β-actin (#4967), phospho-PKCα/βII (#9375), p44/42 MAPK (#9102) or pan-cadherin (#4068) antibody (1∶1000) at 4°C overnight. After washing, the membrane was incubated in anti-biotin HRP-linked antibody (#7075) and HRP-labeled Protein A (#NA9120V) (1∶2000) at room temperature for 1 h, followed by immunodetection with Western Blotting Luminol Reagent (Santa Cruz Biotechnology, Santa Cruz, CA) on Lumi-Imager F1 Workstation (Roche, Basel, Switzerland). The level of β-actin was determined to control the loading amount of total proteins because the antibody for β-actin works very well in the zebrafish. All antibodies were purchased from Cell Signaling Technology while HRP-labeled Protein A was purchased from GE Healthcare.

### Statistical analysis

The mRNA level of each target gene was normalized to the expression of house-keeping gene elongation factor-1α (*ef1a*, now renamed *eef1a1l1*) and expressed as fold change compared with the control group. The statistical analysis was performed with GraphPad Prism 5 (GraphPad Software, San Diego, CA) on Macintosh OS X using one-way ANOVA followed by Newman-Keuls multiple comparison tests. All values were expressed as mean ± SEM. All experiments were repeated at least twice to confirm the results and all treatments were carried out at least in triplicate.

## Results

### Biphasic roles of cAMP-PKA pathway in E2-induced *lhcgr* expression

We have recently reported a strong stimulatory effect of E2 on *lhcgr* expression in zebrafish ovarian follicle cells, which may be mediated by receptors located on the plasma membrane. Interestingly, the response of *lhcgr* occurs in a biphasic manner in a 24-h treatment period with the first major response happening at 3 h followed by a significant drop at 6 h and a rebound at 24 h [10]. This characteristic biphasic response of *lhcgr* expression to E2 treatment *in vitro* suggests distinct action mechanisms of E2 at 3 h (first phase) and 24 h (second phase). To address this issue, we first examined roles of cAMP-PKA pathway in E2 action at 3 h and 24 h because cAMP and PKA have been widely implicated in estrogen signaling in mammalian cells [11]–[17].

**Figure 1:**
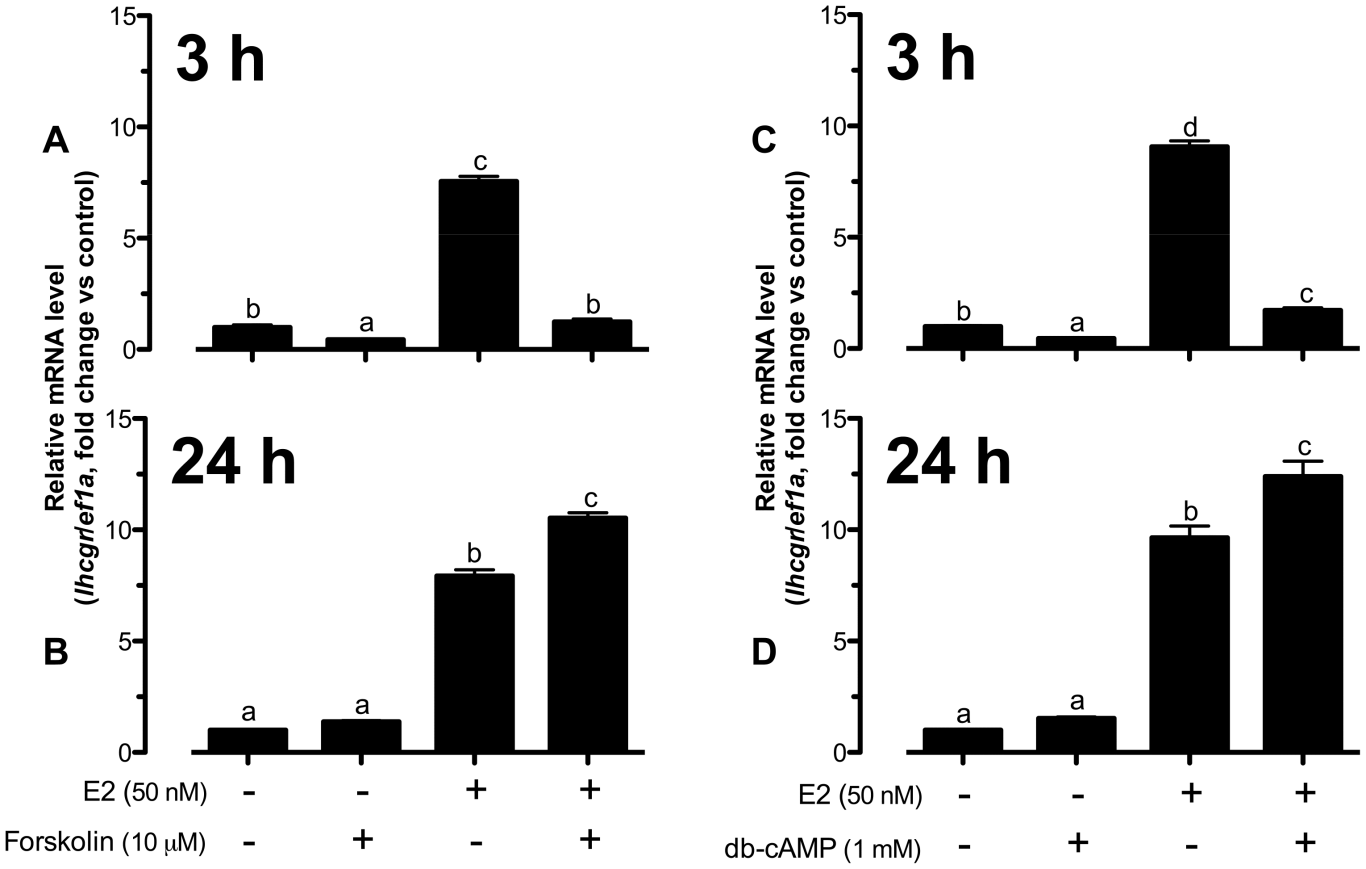
Activation of cAMP pathway modulated E2-stimulated *lhcgr* expression in a biphasic manner. The cells were co-treated with forskolin (10 µM) or db-cAMP (1 mM) and E2 (50 nM) for 3 h (A and C) or 24 h (B and D) before the end of the 24-h treatment period. The follicle cells in each well were lyzed directly in TRI-Reagent for RNA extraction, RT and real-time qPCR to analyze the mRNA levels *lhcgr* and the house-keeping gene *ef1a*. The data were expressed as fold change compared to the control group after normalization to the expression of *ef1a*. Different letters in each data set indicated statistical significance (*P*<0.05; mean ± SEM, *n* = 3–4).

Forskolin, an activator of adenylate cyclase, reduced basal *lhcgr* expression and nearly abolished E2-induced *lhcgr* expression in cultured follicle cells at 3 h of treatment (Fig. 1A). In contrast to the suppressive effect of forskolin at 3 h, 24-h treatment with forskolin slightly enhanced basal and significantly increased E2-stimulated *lhcgr* expression from∼8-fold to∼10-fold compared to the control (Fig. 1B). Similar to forskolin, cAMP analogue db-cAMP also suppressed both basal and E2-induced *lhcgr* expression at 3 h (Fig. 1C) whereas it augmented E2-stimulated *lhcgr* expression from∼9-fold to∼12-fold of expression at 24 h (Fig. 1D).

**Figure 2:**
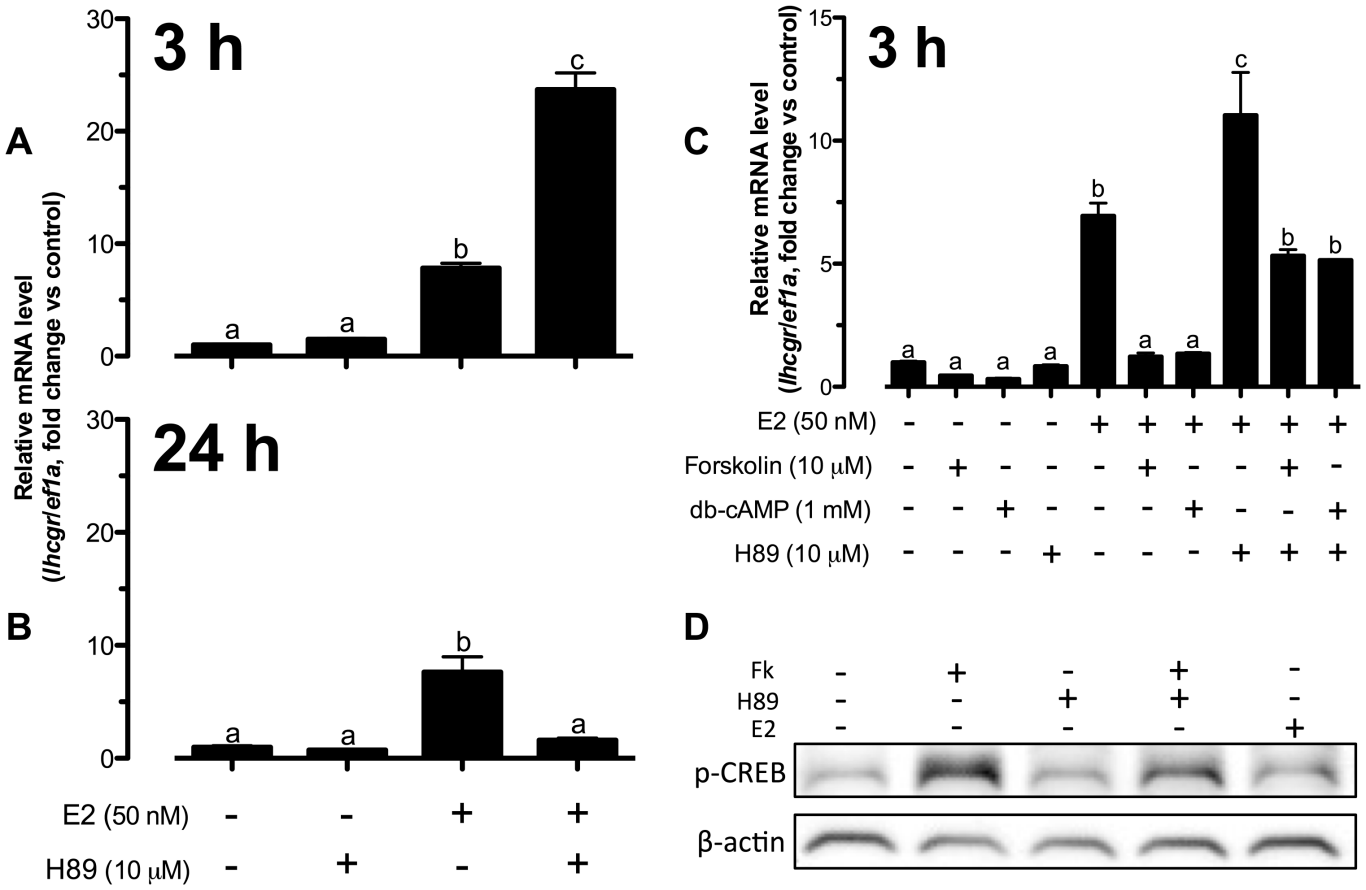
E2-induced *lhcgr* expression was dependent on cAMP-PKA without direct PKA activation in zebrafish follicle cells. (A–C) The cells were pretreated with H89 (10 µM) for 15 min followed by treatment with E2 (50 nM), forskolin (10 µM) and db-cAMP (1 mM) for 3 h or 24 h before the end of the 24-h treatment period. The data were expressed as fold change compared to the control group after normalization to the expression of *ef1a*. Different letters in each data set indicated statistical significance (*P*<0.05; mean ± SEM, *n* = 3–6). (D) The cells were pretreated with H89 (10 µM) for 15 min followed by treatment with E2 (50 nM) and forskolin (10 µM) for 30 min before the end of the 24-h treatment period. Cells were lyzed in SDS sample buffer for Western blot analysis of phospho-CREB (p-CREB) and β-actin expression.

We then investigated if protein kinase A (PKA) played any role in cAMP regulation of *lhcgr* expression. Opposite to the effects of forskolin and db-cAMP, blocking PKA at 3 h with PKA inhibitor H89 slightly but not significantly increased the basal expression of *lhcgr*; however, it synergistically promoted E2-induced *lhcgr* expression from∼8-fold to∼24-fold compared to the control (Fig. 2A). In contrast to the enhancing effects of forskolin and db-cAMP at 24 h, H89 completely eradicated the stimulatory effect of E2 on *lhcgr* at 24 h (Fig. 2B).

To further confirm the role of PKA in cAMP signaling for E2-induced *lhcgr* expression in zebrafish follicle cells, we pretreated the cultured follicle cells with H89 for 15 min followed by a 3-h treatment with forskolin, db-cAMP and E2. In agreement with the result in Fig. 1, forskolin and db-cAMP abolished E2-induced *lhcgr* expression while H89 enhanced it. The pretreatment with H89 completely abolished the inhibitory effects of both forskolin and db-cAMP on E2-induced *lhcgr* expression at 3 h (Fig. 2C); however, the expression of lhcgr was not reversed to the level induced by E2 alone with H89. The partial reversion could be due to the following reasons. First, the relative concentrations of forskolin, db-cAMP and H89 were not optimal for such interactive experiments. An overdose of forskolin and db-cAMP and/or underdose of H89 would likely lead to the incomplete blockade observed. Second, PKA may not be the only signaling molecule downstream of cAMP, and there is a possibility for the involvement of cAMP-dependent but PKA-independent mechanism, such as the cAMP-Epac pathway [18]. This would be an interesting issue to address in the future. The activation of PKA by forskolin was confirmed by its increase of cAMP response element-binding (CREB) protein phosphorylation, which could be reduced by H89. Interestingly, E2 alone had little effect on CREB phosphorylation (Fig. 2D).

### Potential involvement of nuclear estrogen receptors in the biphasic influence of cAMP-PKA on E2-induced *lhcgr* expression

The biphasic effects of cAMP-PKA pathway on E2-induced *lhcgr* expression at 3 h and 24 h raised an interesting question on the involvement of the nuclear estrogen receptors (nERs). To provide clues to this, we examined the effects of forskolin and H89 on the expression of all three nERs in the zebrafish, namely *esr1*, *esr2a* and *esr2b*, in cultured follicle cells.

**Figure 3:**
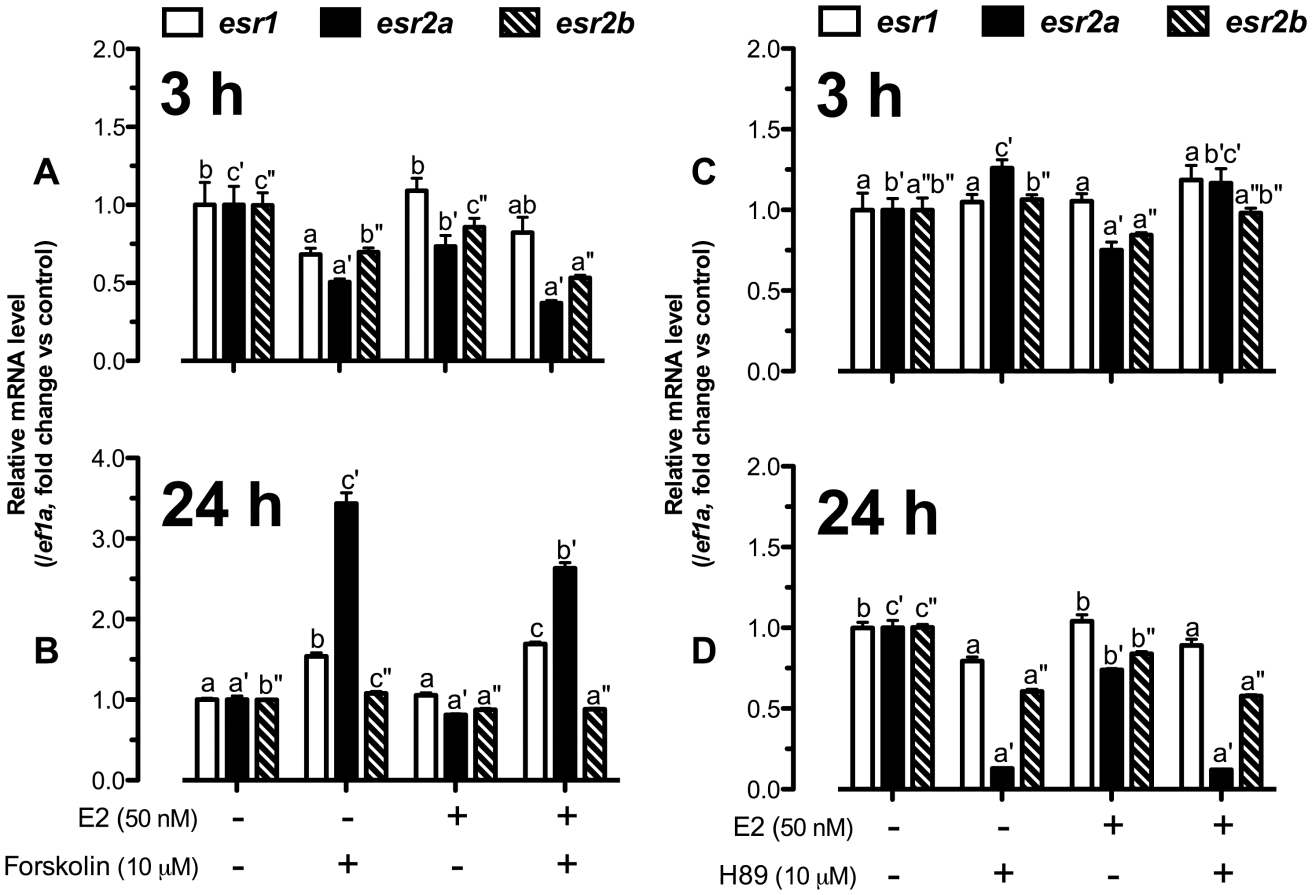
E2 and cAMP-PKA pathway regulated *esr1*, *esr2a* and *esr2b* expression time-dependently. Cultured follicle cells were co-treated with (A and B) forskolin (10 µM) and E2 (50 nM) or pretreated with (C and D) H89 (10 µM) for 15 min followed by treatment with E2 (50 nM) for 3 h or 24 h before the end of the 24-h treatment period. Quantification of mRNA of *esr1*, *esr2a*, *esr2b* and *ef1a* was carried out. The data were expressed as fold change compared to the control group after normalization to the expression of *ef1a*. Different letters in each data set indicated statistical significance (*P*<0.05; mean ± SEM, *n* = 4).

As shown in Fig. 3A, forskolin at 3 h reduced the expression of all three receptors in the presence or absence of E2 with the response of *esr2a* being the most prominent. E2 also slightly decreased *esr2a* expression in the presence or absence of forskolin. On the contrary, after 24-h treatment, forskolin significantly increased *esr1* and *esr2a* expression and the effect on *esr2a* was slightly but significantly reduced by E2 (Fig. 3B).

In contrast to the effect of forskolin (Fig. 3A), treatment of the follicle cells with H89 for 3 h slightly but significantly stimulated *esr2a* expression (Fig. 3C); however, H89 significantly suppressed the expression of all three nERs, especially *esr2a,* at 24 h of treatment (Fig. 3D). Notably, *esr2a* was the most responsive gene to both forskolin and H89 among three nERs (Fig 3A–D).

**Figure 4:**
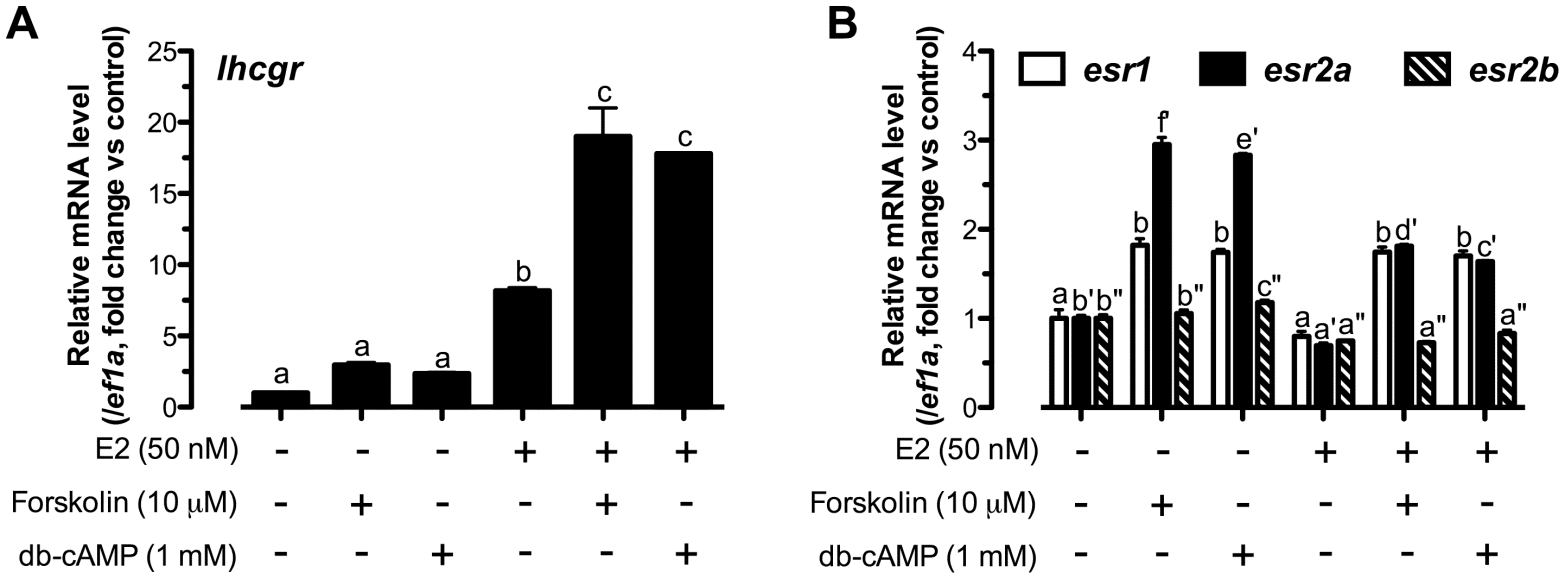
Pre-activation of cAMP enhanced E2-stimulated *lhcgr* expression likely by promoting follicle cell responsiveness to E2. Cells were administered with forskolin (10 µM) or db-cAMP (1 mM) for 24 h before a 3-h treatment of E2 (50 nM). Relative mRNA levels of (A) *lhcgr*, (B) *esr1*, *esr2a* and *esr2b* were expressed as fold change compared to the control group after normalization to the expression of *ef1a*. Different letters in each data set indicated statistical significance (*P*<0.05; mean ± SEM, *n* = 3).

To test the idea that the enhancing effect of cAMP-PKA pathway at 24 h on E2-induced *lhcgr* expression was due to the increased expression of nERs, in particular *esr2a*, we performed an experiment by pretreating the follicle cells with forskolin and db-cAMP for 24 h followed by a 3-h treatment with E2. If the effects of forskolin and db-cAMP on nER expression were reflected at the protein level, we would expect that a 24-h pretreatment with forskolin or db-cAMP would influence the responsiveness of follicle cells to E2 and therefore its stimulation of *lhcgr* expression. As shown in Fig. 4A, E2 stimulated *lhcgr* expression as expected whereas pretreatment with forskolin or db-cAMP both synergistically enhanced the stimulatory effect of E2 on *lhcgr* from approximately 8-fold to 18-fold. This was in sharp contrast to the inhibitory effects of forskolin and db-cAMP on E2-induced *lhcgr* expression at 3 h when the cells were co-treated with E2 and either forskolin or db-cAMP for the same time (3 h). Meanwhile, the pretreatment with forskolin and db-cAMP significantly increased both *esr1* and *esr2a* expression while E2 suppressed forskolin- or db-cAMP-induced *esr2a* but not *esr1* expression (Fig. 4B), which agreed with the result shown in Fig. 3B. Similar to that shown in Fig. 3, *esr2a* was the most responsive nER to forskolin and db-cAMP compared with *esr1* and *esr2b*.

### Influence of PKC pathway on E2-induced *lhcgr* expression

**Figure 5:**
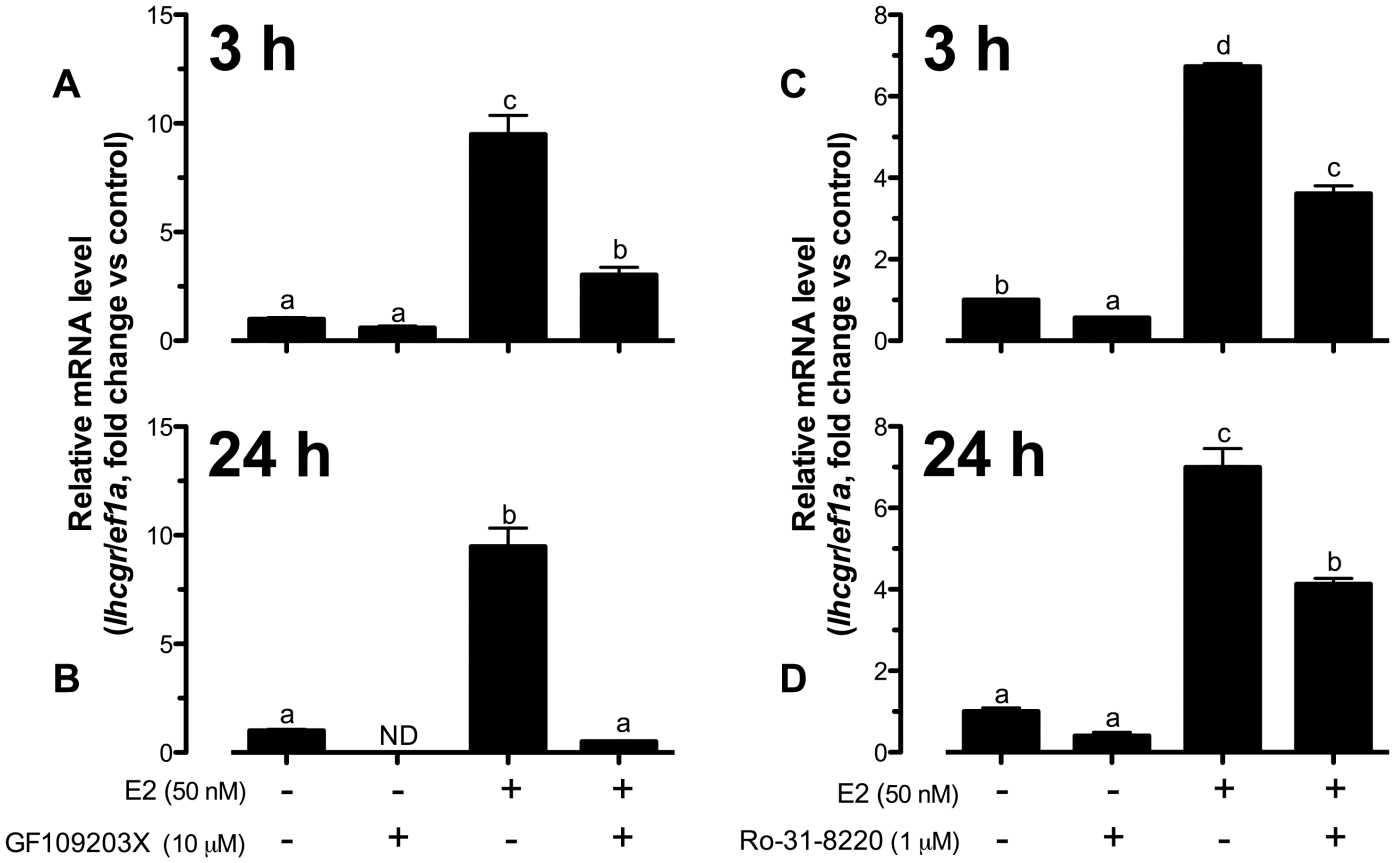
Both basal and E2-induced *lhcgr* expression were highly dependent on PKC pathway. Effect of GF109203X (A and B) and Ro-31-8220 (C and D) on basal and E2-stimulated *lhcgr* expression at 3 h and 24 h of treatment in cultured zebrafish follicle cells. The cells were pretreated with GF109203X (10 µM) or Ro-31-8220 (1 µM) for 15 min followed by treatment with E2 (50 nM) for 3 h or 24 h before the end of the 24-h treatment period. The data were expressed as fold change compared to the control group after normalization to the expression of *ef1a*. Different letters in each data set indicated statistical significance (*P*<0.05; mean ± SEM, *n* = 3–4).

Having shown the importance of cAMP-PKA pathway in E2-induced *lhcgr* expression, we turned our attention to protein kinase C (PKC), another signaling pathway that has been reported to play a role in E2 signaling [11], [12], [14], [19]. At 3-h of treatment, the PKC inhibitor GF109203X (added 15 min earlier) significantly suppressed E2-stimulated *lhcgr* expression from∼9-fold to∼3-fold (Fig. 5A) and it nearly abolished the effect of E2 at 24 h (Fig. 5B), which was in contrast to the biphasic effects of cAMP-PKA at the two time points. GF109203X also affected the basal *lhcgr* expression at both 3 and 24 h. It reduced the basal level albeit insignificantly at 3 h and the expression turned undetectable at 24 h (Fig. 5A and B). To further confirm the role of PKC, we tested another PKC inhibitor Ro-31-8220. Similarly, Ro-31-8220 reduced basal and E2-stimulated *lhcgr* expression at both 3-h and 24-h treatment; however, its potency was not as high as that of GF109203X (Fig. 5C and D).

**Figure 6:**
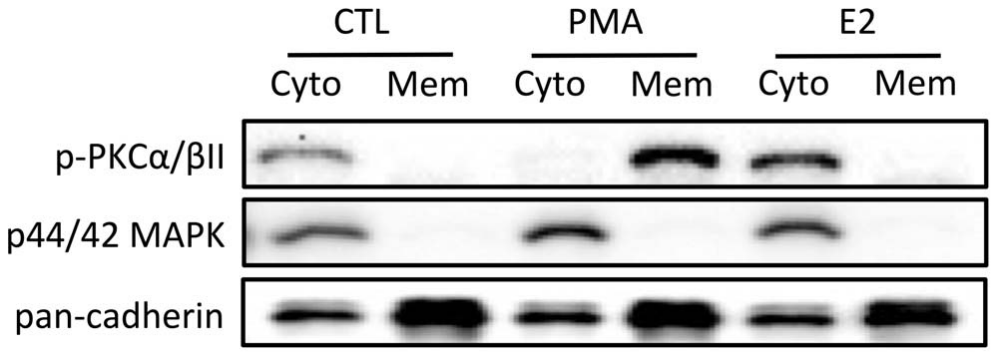
E2 could not directly activate PKC in zebrafish cultured follicle cells. The cells were treated with PKC activator, PMA (100 nM) or E2 (50 nM) for 20 min before the end of the 24-h treatment period. The treated cells were fractionated into cytosol (Cyto) and membrane (Mem) protein fractions followed by SDS-PAGE and Western blot analysis against phospho-PKCα/βII (p-PKCα/βII), p44/42 MAPK (cytosol marker) and pan-cadherin (membrane marker).

The strong dependence of E2-stimulated *lhcgr* expression on PKC pathway led us to speculate whether E2 could directly stimulate the PKC pathway to increase *lhcgr* expression in the zebrafish ovary. To explore this possibility, we examined membrane translocation of PKC after E2 treatment as PKC activation is associated with its translocation from the cytosol to the plasma membrane [20], [21]. As expected, PKC activator PMA induced a clear translocation of p-PKCα/βII from cytosol to plasma membrane. However, similar to the control, p-PKCα/βII remained in the cytosol fraction after E2 treatment while both PMA and E2 seemed to increase p-PKCα/βII abundance (Fig. 6).

### PKC regulation of estrogen receptor expression

Although E2-induced *lhcgr* expression was highly dependent on PKC pathway, E2 did not seem to activate PKC directly as shown in Fig. 6. One possible mechanism of the strong modulatory effect of PKC on E2 could be the change of nER expression, which would in turn influence the responsiveness of follicle cells to E2. To test this hypothesis, we examined the expression of *esr1*, *esr2a* and *esr2b* in the presence of GF109203X or Ro-31-8220 at 3 h and 24 h.

**Figure 7:**
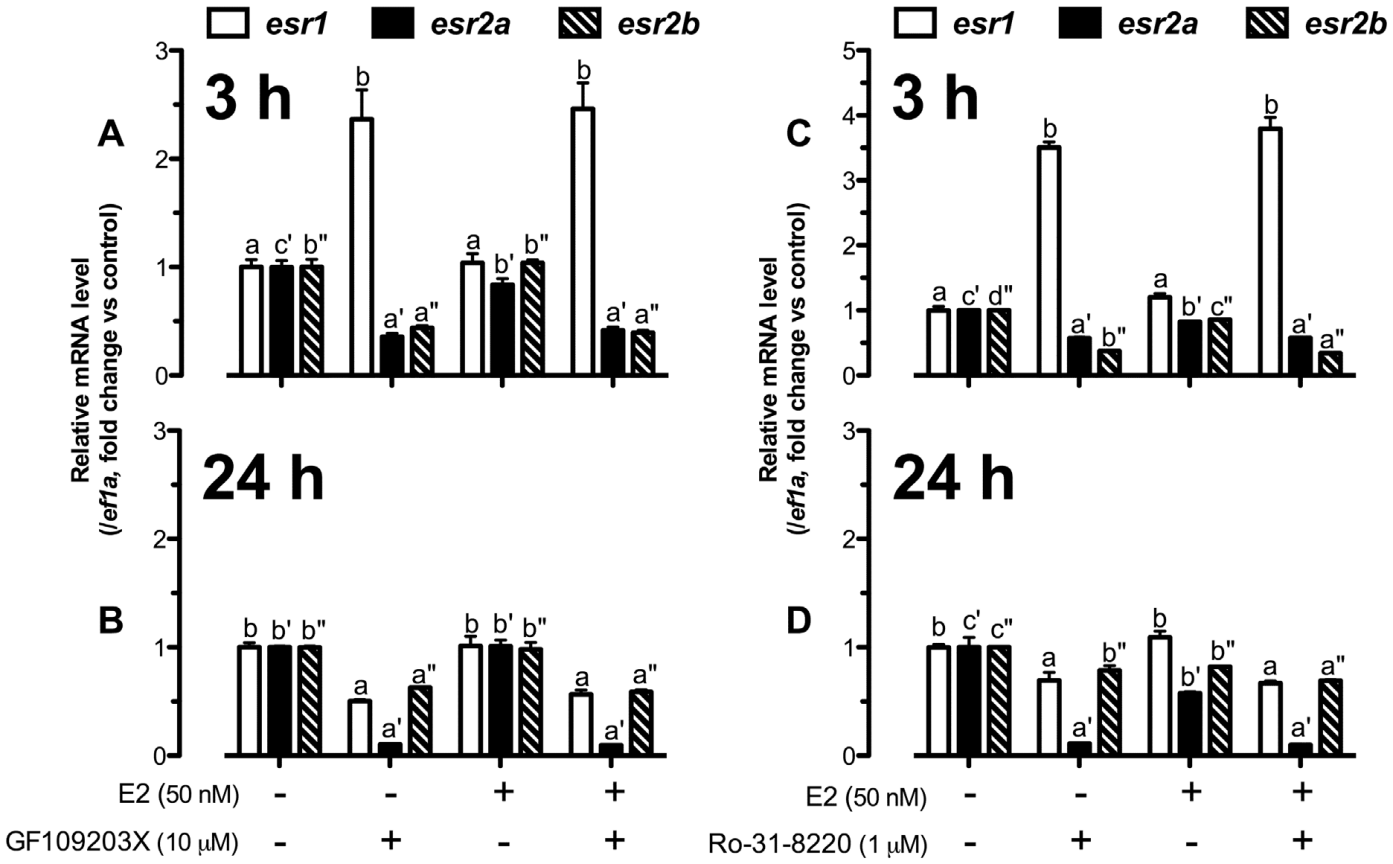
PKC pathway was crucial for nuclear estrogen receptor expression. The cultured follicle cells were pretreated with GF109203X (10 µM) or Ro-31-8220 (1 µM) for 15 min followed by treatment with E2 (50 nM) for 3 h (A and C) or 24 h (B and D) before the end of the 24-h treatment period. Quantification of mRNA level of *esr1*, *esr2a* and *esr2b* was carried out. The data were expressed as fold change compared to the control group after normalization to the expression of *ef1a*. Different letters in each data set indicated statistical significance (*P*<0.05; mean ± SEM, *n* = 3–4).

As shown in Fig. 7A, GF109203X significantly increased *esr1* expression to approximately 2.5-fold, but suppressed the expression of both *esr2a* and *esr2b* to∼0.4-fold at 3 h in the presence or absence of E2. At 24 h, however, GF109203X suppressed the expression of all three nERs. The expression of *esr1* decreased in contrast to its increase at 3 h, and the expression of *esr2a* expression further decreased to nearly undetectable level. Again, E2 had no effect on GF109203X-induced response of any nER (Fig. 7B). In agreement with GF109203X, another PKC inhibitor Ro-31-8220 also stimulated *esr1* but reduced *esr2a* and *esr2b* expression at 3 h (Fig. 7C) while it tended to suppress all three nERs, especially *esr2a*, at 24 h (Fig. 7D).

## Discussion

There has been increasing evidence for E2 regulation of GTHR expression in the ovary of teleosts. Injecting black porgy with E2 stimulated the expression of both *fshr* and *lhcgr* [7]. In the coho salmon, long-term treatment with FSH *in vitro* elevated E2 production before the rise of *lhcgr* expression [5], suggesting a possible mediating role for E2 in regulating *lhcgr* expression. Recently, we have also demonstrated a potent stimulatory effect of E2 on GTHR expression in the zebrafish ovary, in particular *lhcgr*. Interestingly, the time-course of *lhcgr* expression in response to E2 during 24-h *in vitro* treatment showed a distinct biphasic pattern, consisting of an acute increase at 1.5 to 3 h of treatment and a second increase at 24 h after a declining phase [10]. Despite these studies in teleosts, the underlying mechanisms of E2 signaling in regulating GTHRs remain largely unknown. In the zebrafish, we have shown that the E2 stimulation of *lhcgr* expression was mediated via nERs; however, these nuclear receptors seemed to be located on the plasma membrane [10]. This raises a question on the intracellular signaling mechanism underlying E2 action, in particular the biphasic response of *lhcgr*. The evidence for membrane location of nERs points to the possibility that classical intracellular signal transduction pathways might be involved in mediating or modulating E2 signaling.

In mammals, mounting evidence has demonstrated membrane-bound nERs and their involvement in E2 actions [22]. The presence of these classical nERs on the plasma membrane has been demonstrated in various cell types by using E2-BSA (plasma membrane-impermeable form of E2) and ICI 182,780 (nER antagonist) [16], [23], [24]. Using specific antibodies and E2-BSA-FITC, the expression of nERs on the plasma membrane has been visualized [17], [24], [25]. Strong evidence for the translocation of nERs to the plasma membrane further confirms the existence of nERs on the plasma membrane [26]–[30]. These membrane-anchored ERs (mERs) are involved in rapid signal transduction activated by E2, which can lead to non-genomic effects [30]–[32].

There has been evidence that E2 can activate various signal transduction pathways. E2 activation of MAPK3/1 has been reported in the brain cells [13], [31], [33], skeletal muscle myoblasts [34], Sertoli cells [27], adipocytes [16], endothelial cells [17], [24] and cancer cell lines [15], [30]. Another major signaling pathway activated by E2 is cAMP-PKA, which has been reported in the brain cells [11]–[14], cancer cells [15], adipocytes [16] and endothelial cells [17]. In addition, PKC is also well documented to mediate E2 signals in neurons [11], [12], [14], [19]. Yet another well established signaling pathway activated by E2 is the PI3K-Akt pathway, which has been shown in the brain cells [31], uterus luminal epithelial cells [35], endothelial cells [17], adipocytes [16] and cancer cells [25], [36], [37]. Furthermore, E2 has been reported to suppress the hypoxia-induced p38 MAPK phosphorylation in hepatocytes [38] but stimulate p38 MAPK in colon cancer cells [39].

Although limited in teleosts, there have been several studies on E2 signaling, in particular the involvement of classical intracellular signaling pathways. In the zebrafish oocytes, E2 stimulates the production of cAMP by binding to its G protein-coupled estrogen receptor 1 (Gper) [40]. The cAMP production is also increased in HEK293 cells transfected with Atlantic croaker Gper [41]. In addition, E2 actions involve cAMP-PKA and PKC pathways in the brain of catfish [42], [43] while MAPK3/1 phosphorylation is increased by E2 in rainbow trout hepatocytes [44].

In the zebrafish ovary, despite the lack of evidence for a direct E2 stimulation of the cAMP-PKA pathway in the follicle cells, our experiments in the present study clearly showed an important role for cAMP-PKA pathway in modulating E2-stimulated *lhcgr* expression. Interestingly, the cAMP-PKA pathway appeared to play different roles in the biphasic response of *lhcgr* to E2, *i.e.*, the acute action at 3 h and chronic effect at 24 h. Activating the cAMP-PKA pathway abolished the stimulatory effect of E2 on *lhcgr* at 3 h, suggesting a powerful inhibitory influence of cAMP-PKA pathway on the signaling of E2 in its acute regulation of *lhcgr* expression. In contrast, the role of cAMP-PKA in E2-stimulated *lhcgr* expression reversed at 24 h; it enhanced E2 stimulation of *lhcgr* at this time point. This result suggests an essential role for cAMP-PKA in the long-term stimulatory effect of E2 on *lhcgr*. Taken together, the inhibitory influence of cAMP-PKA pathway on the short-term effect of E2 on *lhcgr* expression and its stimulatory influence on the long-term E2 effect suggest an intricate time-dependent gating role for cAMP-PKA pathway in the biphasic response of *lhcgr* expression to E2 treatment.

The gating role of cAMP pathway in signal transduction has been widely reported in mammals [45]. PKA gates the EGF-induced MAPK3/1 activation by inhibiting Raf-1 in rat fibroblasts [46]. The activation of cAMP-PKA pathway also obstructs the Ras-induced transformation of mouse embryonic cell line NIH 3T3 cells [47]. Recently, a time-dependent gating role of cAMP pathway has been reported in the suprachiasmatic circadian clock of rats [48]. Activation of cAMP-PKA pathway enhances glutamate-induced rhythm changes in suprachiasmatic neuronal activity at early night while inhibits that at late night. This study, together with our current results and the existence of peripheral circadian clocks in the zebrafish [49], has raised an interesting question on whether the dual roles of cAMP-PKA pathway in E2-stimulated *lhcgr* expression involve circadian control in the zebrafish ovary.

In teleosts, previous studies on Gper have reported an E2-stimulated cAMP production in Atlantic croaker and zebrafish [40], [41]. E2 or Gper agonist G1 signals through Gper exclusively expressed on the oocyte plasma membrane to inhibit oocyte maturation in the zebrafish ovary [40]. As there is no *gper* expression in the zebrafish follicle cells [10], [40], [50], the cAMP-PKA pathway in these cells is obviously Gper-independent.

In mammals, E2 increases cAMP production in mice endothelial cells [17], and PKA is involved in a wide range of E2 activities in different cell types including the adipocytes [16], adrenal medulla cancer cells [15], GnRH neurons [11], and other brain cells in the hypothalamus [12], cerebellum [13] and hippocampus [14]. However, our results showed that E2 itself did not alter the abundance of pCREB, suggesting that E2 could not directly activate or inhibit cAMP-PKA pathway. Therefore, the cAMP-PKA pathway is unlikely involved in mediating E2 action; however, it strongly modulates or gates E2 actions in its regulation of *lhcgr* expression.

Since the positive gating effect of cAMP-PKA pathway took 24 h to occur, it is conceivable that this effect might involve a secondary mechanism that indirectly modulates E2 signaling on *lhcgr* expression. One such mechanism could be a change in the responsiveness of the follicle cells to E2. This idea was tested and supported by the data in the present study. Treatment of cultured follicle cells with forskolin for 24 h significantly increased both *esr1* and *esr2a* expression while 24-h inhibition of PKA by H89 strongly suppressed the expression of all three nER subtypes. Therefore, the cAMP-PKA pathway seems to be crucial for maintaining follicle cell responsiveness to E2 as the expression of *esr1* and *esr2a* was dependent on this pathway in the zebrafish ovary, particularly *esr2a*. This mechanism was further confirmed by the potentiation of E2 action at 3 h after 24-h pretreatment with forskolin and db-cAMP. In contrast to our observation in the zebrafish, 8-bromo-cAMP (cAMP analogue) and forskolin down-regulated both ERα and ERβ in cultured human granulosa-luteal cells after a long-term 24-h treatment [51], [52], highlighting a possible discrepancy of E2 signaling between different species, cell types, or experimental conditions.

In agreement with the negative gating role of cAMP-PKA pathway at 3 h, the increase in cAMP production at this time point slightly suppressed instead of increasing nER expression. The weak response of nER expression to cAMP-PKA in short-term treatment indicates that the level of nER expression was unlikely a major mechanism for cAMP-PKA blockade of E2-induced *lhcgr* expression at 3 h. The exact mechanism by which cAMP-PKA suppresses E2 action remains unknown. Interestingly, *esr2a* was the most responsive nER subtype among the three nERs to cAMP-PKA regulation at both 3 h and 24 h. The high responsiveness of *esr2a* expression to regulation suggests that Esr2a may play a major role in mediating E2 stimulation of *lhcgr* expression in the zebrafish ovary.

In addition to forskolin and H89, E2 itself also regulated nER subtype expression. It down-regulated basal *esr2a* expression at 3 h and forskolin-induced *esr2a* expression at 24 h with little effects on the other two subtypes. This homologous regulation has also been reported in other teleosts despite varying responses in different species and tissues. In the goldfish, E2 induced expression of nERs in both gonads [53] and liver [53]–[55]. However, another study in the goldfish reported no effect of E2 on *esr2a* and *esr2b* expression in the liver and testis [56], which could be due to different doses used. A recent study on zebrafish hepatocytes revealed stimulatory and inhibitory effect of E2 on *esr1* and *esr2a* expression at 48 h, respectively [57]. The short-term homologous down-regulation of nERs, particularly *esr2a*, by E2 may serve as a negative feedback mechanism to control E2 signaling in the zebrafish follicle cells. This negative feedback mechanism may also account for the decline of E2-stimulated *lhcgr* expression at 6 h of treatment [10].

In addition to cAMP-PKA pathway, E2 signaling may also involve PKC pathway. E2 has been well documented to activate PKC in the brain cells of mammals, for example, mice [11], guinea pigs [12] and rats [14], [19]. In the current study, we also demonstrated a potential role for PKC in E2-stimulated *lhcgr* expression. The presence of GF109203X, a PKC inhibitor, significantly reduced the E2-stimulated *lhcgr* expression at 3 h and longer treatment for 24 h nearly abolished the effect of E2. Similar effects were also observed with Ro-31-8220, another PKC inhibitor, although it was not as potent as GF109203X. The action of PKC appeared to be different from that of PKA in that blockade of PKA enhanced the acute E2 effect at 3 h but suppressed its long-term effect at 24 h. Despite the strong influence of the PKC pathway, it might not be the one that mediated E2 action as E2 failed to activate PKC as evidenced by the lack of p-PKCα/βII translocation in response to E2. However, we cannot exclude the possibility that other PKC isozymes might be activated by E2, and this would be an interesting issue to investigate in the future. Interestingly, E2 tended to mimic PMA to increase the level of PKC. Whether this change plays a role in *lhcgr* expression remains to be elucidated. In contrast with the dual actions of cAMP-PKA pathway, PKC pathway appears to play a consistently positive gating role in E2-stimulated *lhcgr* expression during 24-h period.

Similar to the cAMP-PKA pathway, PKC pathway was also involved in regulating the expression of nERs in the zebrafish follicle cells as reported in mammalian models. In human breast cancer cells [58]–[61], increased PKC activity down-regulated nERs. Similar inverse relationship has also been reported in the bone cells [62]–[64], granulosa cells [52], [65], and uterus [66]. On the contrary, activating PKC in skeletal muscle myoblasts of mice enhanced E2-stimulated ERα expression [34]. In the present study, consistent with the positive gating role of PKC pathway in E2-stimulated *lhcgr* expression at both 3 h and 24 h, inhibiting PKC by GF109203X and Ro-31-8220 strongly down-regulated the expression of *esr2a* and *esr2b* at 3 h and all three nERs at 24 h whereas both inhibitors significantly augmented *esr1* expression at 3 h. These results indicate that PKC likely modulates E2 effect on *lhcgr* by differentially regulating nER expression, which would in turn influence the responsiveness of the follicle cells to E2. Interestingly, the significant decrease of *esr2a* expression to PKC inhibitors at 24 h is in agreement with its high responsiveness to the cAMP-PKA activity, which suggests again that Esr2a might be a major nER mediating E2 regulation of *lhcgr* expression. In addition, the short-term stimulation of *esr1* expression by both PKC inhibitors indicates that the three nERs are likely subject to differential regulation in the zebrafish ovary.

Although our data on PKA and PKC both suggest important roles for nERs in their modulation of E2 signaling to regulate *lhcgr* expression, the evidence remains indirect as it is based on correlation of gene expression. More direct evidence could be obtained by such approaches as gene knockdown with siRNA or morpholino; however, the zebrafish follicle cells in primary culture are extremely difficult to transfect, making this approach unfeasible at this moment. The recent emergence of gene knockout technology in the zebrafish using transcription activator-like effector nuclease (TALEN) promises to provide an alternative platform to understand functions of individual genes in this model, which would help provide definitive evidence for the importance of each nER isoform in the zebrafish ovary, including their roles in E2 signaling as well as PKA and PKC modulation of the signaling.

In addition to PKA and PKC pathways on *lhcgr* expression, other signaling pathways may also play a role in cultured zebrafish follicle cells. Activation of cAMP-PKA pathway by forskolin or db-cAMP could not completely suppress E2-induced *lhcgr* expression down to the basal level. Furthermore, E2 could raise the undetectable level of GF109203X-inhibited *lhcgr* expression back to the level comparable with the control. These results suggest that there may be other signaling pathways that also influence E2-stimulated *lhcgr* expression. We have recently reported that stimulation of p38 MAPK activity greatly enhances the performance of E2 in regulating *lhcgr* expression while MEK-MAPK3/1 pathway likely plays a permissive role in the regulation [10]. Moreover, our recent data also suggest a role for PI3K-Akt pathway in the process (data not shown). Together with the current study, these pieces of evidence point to the involvement of multiple classical signal transduction pathways in E2-stimulated *lhcgr* expression; however, most of these pathways likely modulate or gate but not directly mediate E2 effect.

The PKA and PKC pathways represent two major signaling pathways activated by GTHRs. Upon binding by FSH and LH, FSHR and LHCGR activate G_αs_ protein to increase intracellular cAMP level [67]–[71] to activate PKA, which in turn modulates target gene transcription through CREB [71]–[73]. Meanwhile, phospholipase C (PLC) is also activated by FSH and LH to hydrolyze phosphatidylinositol-4,5-bisphosphate (PIP_2_) into inositol-1,4,5-triphosphate (IP_3_) and diacylglycerol (DAG), which mobilizes calcium ions (Ca^2+^) and activates PKC, respectively [73], [74]. Our current study, therefore, suggests a possibility of negative feedback and homologous regulation of *lhcgr* by LH, which may activate these signaling pathways to gate the E2 action on the expression of its own receptor. A recent study on human granulosa cells reveals that LH/hCG binds to LHCGR to activate cAMP-PKA pathway to down-regulate LHCGR [75]. Whether cAMP-PKA pathway mediates LH signal to inhibit *lhcgr* expression in the zebrafish ovary is unknown and will be investigated in the future.

**Figure 8:**
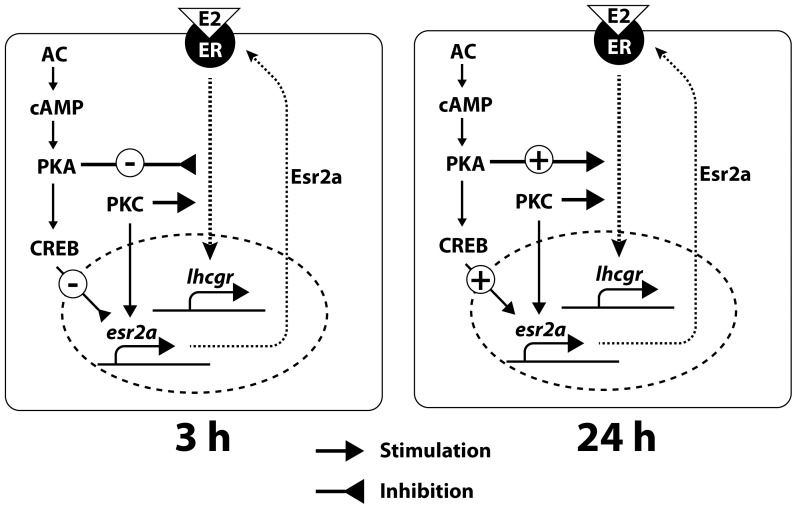
Hypothetical model showing the biphasic signaling pathways in E2-stimulated *lhcgr* expression in the zebrafish ovary. AC, adenylate cyclase; PKA, protein kinase A; PKC, protein kinase C; CREB, cAMP-responsive element binding protein.

In summary, although we did not identify any direct mediators of E2 signaling downstream of its receptors, especially the ones responsible for the biphasic *lhcgr* response to E2, the current study demonstrated differential gating roles of cAMP-PKA and PKC pathways (Fig. 8). The gating role of cAMP-PKA pathway appeared to be time-dependent, which negatively modulated E2-stimulated *lhcgr* expression in short-term (3 h) but promoted it in long-term (24 h). The long-term effect was likely mediated by up-regulating *esr2a* to enhance E2 responsiveness of zebrafish follicle cells. In contrast, PKC pathway exerted a consistently positive gating role in E2-induced *lhcgr* expression, which also appeared to involve regulating the expression of *esr2a* to some extent. The present study provides strong evidence for the involvement of multiple signaling pathways in E2 stimulation of *lhcgr* expression in the zebrafish ovary. The exact roles and interactions of these pathways will be interesting issues for future studies.
